# Vagus nerve stimulation parameters evoke differential neuronal responses in the locus coeruleus

**DOI:** 10.14814/phy2.15633

**Published:** 2023-03-10

**Authors:** Ariana Farrand, Vincent Jacquemet, Ryan Verner, Misty Owens, Eric Beaumont

**Affiliations:** ^1^ Department of Biomedical Sciences Quillen College of Medicine, East Tennessee State University Johnson City Tennessee USA; ^2^ Department of Pharmacology and Physiology Institute of Biomedical Engineering, University of Montreal Montreal Quebec Canada; ^3^ Research Center Sacred Heart Hospital of Montreal Montreal Quebec Canada; ^4^ Neuromodulation Division LivaNova PLC Houston Texas USA

**Keywords:** bursting paradigm, in vivo electrophysiology, locus coeruleus, neural activity, neuronal synchrony, vagus nerve stimulation

## Abstract

Vagus nerve stimulation (VNS) is used to treat drug‐resistant epilepsy and depression, with additional applications under investigation. The noradrenergic center locus coeruleus (LC) is vital for VNS effects; however, the impact of varying stimulation parameters on LC activation is poorly understood. This study characterized LC activation across VNS parameters. Extracellular activity was recorded in rats' left LC while 11 VNS paradigms, utilizing variable frequencies and bursting characteristics, were pseudorandomly delivered to the left cervical vagus for five cycles. Neurons' change from baseline firing rate and timing response profiles were assessed. The proportion of neurons categorized as responders over 5 VNS cycles doubled in comparison to the first VNS cycle (*p* < 0.001) for all VNS paradigms, demonstrating an amplification effect. The percentage of positively consistent/positive responders increased for standard VNS paradigms with frequencies ≥10 Hz and for bursting paradigms with shorter interburst intervals and more pulses per burst. The synchrony between pairs of LC neurons increased during bursting VNS but not standard paradigms. Also, the probability of evoking a direct response during bursting VNS was higher with longer interburst intervals and a higher number of pulses per burst. Standard paradigms between 10–30 Hz best positively activates LC with consistency to VNS while the best bursting paradigm to increase activity was 300 Hz, seven pulses per burst separated by 1 s. Bursting VNS was effective in increasing synchrony between pairs of neurons, suggesting a common network recruitment originating from vagal afferents. These results indicate differential activation of LC neurons depending on the VNS parameters delivered.

## Graphical abstract

The percentage of positively consistent/positive responders increased for standard VNS paradigms with frequencies ≥ 10Hz and for bursting paradigms with shorter interburst intervals and more pulses per burst. Bursting VNS was effective in increasing synchrony between pairs of neurons, suggesting a common network recruitment originating from vagal afferents. These results indicate differential activation of LC neurons depending on the VNS parameters delivered.

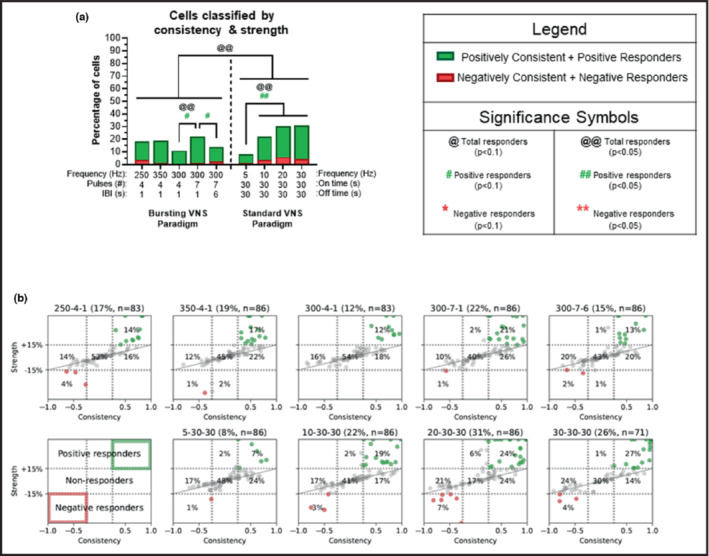

## 1 INTRODUCTION

Vagus nerve stimulation (VNS) is currently approved to treat drug‐resistant epilepsy and difficult‐to‐treat depression. Implantable VNS devices have been proven safe and effective for these disorders (Beekwilder & Beems, 2010). Additionally, there is preclinical evidence that VNS may be effective in a wide range of disorders including heart failure, rheumatoid arthritis, migraine, and Parkinson's disease (De Couck et al., 2014; Farrand et al., 2020; Schwartz et al., 2008; Yuan & Silberstein, 2015a). Despite the vast availability of literature regarding the mechanisms of action for VNS (Johnson & Wilson, 2018; Vonck & Larsen, 2018; Yuan & Silberstein, 2015b), the role and magnitude of these mechanisms' contributions to specific therapeutic effects remains poorly understood.

Activation of the vagus nerve both acutely and chronically leads to upregulation of cell activation markers in brain regions receiving vagal innervation along with their upstream targets (Cunningham et al., 2008). One key nucleus associated with VNS effects in the brain is the noradrenergic center locus coeruleus (LC). It receives indirect innervation from the vagus nerve via the solitary nucleus (Fornai et al., 2011), as well as projections from the paragigantocellular nucleus, also innervated by the solitary nucleus (Ruffoli et al., 2011). Increased firing rates of LC neurons during VNS have been correlated to chronic treatment duration, leading to higher levels of norepinephrine release in LC target regions (Biggio et al., 2009; Dorr & Debonnel, 2006). Anti‐depressant and anti‐epileptic effects of VNS can be blocked with LC lesions, demonstrating the necessity of this nucleus for VNS effects in the brain (Furmaga et al., 2011; Grimonprez et al., 2015). Modulation of LC activity by VNS occurs more rapidly than other brain regions such as the raphe, further indicating that VNS effects in the brain are likely mediated via the LC (Dorr & Debonnel, 2006).

The LC regulates several forebrain networks using phasic response profiles to drive plasticity, indicating the importance for timing of LC responses (Borland et al., 2016; Sara & Bouret, 2012; Schwarz & Luo, 2015). Indeed, small clusters of highly synchronous and target‐specific LC neurons have been correlated to differential forebrain targets (Hirschberg et al., 2017; Totah et al., 2018). Consistency of LC firing to the type of VNS input delivered can indicate activation of discrete LC pathways, and synchrony of LC neurons with each other indicates activation of these clusters.

VNS effects are typically attributed to activation of myelinated vagal A‐type afferents that project to the solitary nucleus and from there to higher brain regions including the LC (McAllen et al., 2018). Low VNS frequencies (1–10 Hz) are thought to be particularly effective to activate systemic anti‐inflammatory pathways, most likely attributed to the direct vagal efferent tone during VNS, as shown in rheumatoid arthritis and gut motility work (Koopman et al., 2016; Lu et al., 2018). However, similar parameters have limited efficacy for the central nervous system (Farrand et al., 2020; Giordano et al., 2017; Müller et al., 2013; Olejniczak et al., 2001). Moderate frequencies (20–30 Hz) are used to treat epilepsy and depression, and as little as a 10% duty cycle is effective clinically (Rush et al., 2005; Sackeim et al., 2007; Yamamoto, 2015). Bursting VNS has been investigated as alternative stimulation for epilepsy. Studies in preclinical rodents and dogs suggest bursting may be more effective than 20–30 Hz clinical parameters for epilepsy and Parkinson's disease (Alexander & McNamara, 2012; Farrand et al., 2020; Martlé et al., 2014). Bursting VNS is thought to be effective because it reflects natural rhythms of phasic LC activation, leading to elevated evoked potentials in LC targets (Ito & Craig, 2005; Miguelez et al., 2011). Therefore, the goal of this study was to examine the response profile of LC neurons under variable VNS conditions to determine how to optimize activation of this nucleus.

## 2 MATERIALS AND METHODS

### 2.1 Animals

Adult male Sprague Dawley rats (Envigo) weighing 360 ± 22 g were used for all experiments (*n* = 12). Rats were pair‐housed in an AAALAC‐accredited facility at East Tennessee State University (ETSU) under a 12 h normal light: dark cycle with free access to food and water. This study and all procedures were approved by the University Committee on Animal Care at ETSU (study P211101) and comply with the NIH Guide for Care and Use of Laboratory Animals.

### 2.2 Surgical preparations

Rats were deeply anesthetized using 5% isoflurane (Piramal Healthcare) then maintained at 2%–3% isoflurane until surgical preparations were complete. Surgical procedures were conducted as previously described (Beaumont et al., 2017). Briefly, the jugular vein was cannulated for administration of α‐chloralose (Sigma), and a bipolar fusion nerve cuff (Micro‐Leads, FNC‐500‐V‐R‐2C‐30) was implanted around the left cervical vagus nerve. The trachea was cannulated for mechanical ventilation at 60 breaths/min with sustained 3% expired carbon dioxide (monitored via CWE CAPSTAR‐100). Rats were then fixed in a stereotaxic frame with the nose tilted downward at a 15° angle for better access to the LC nucleus. The skull surface was exposed, and a burr hole was made using a micromotor drill (Foredom) at 9.5 mm caudal to bregma and 1.1 mm left of midline (Paxinos & Watson, 2005). Anesthesia was then switched to α‐chloralose beginning with a bolus injection (50 mg/kg iv) given over 20 min, followed by cessation of isoflurane and continuous α‐chloralose infusion to maintain anesthesia (20 mg/kg·h iv). Rats were stabilized for 30 min before initiating neural recordings. Heart rate was monitored throughout all experiments (Grass Instrument P511) and body temperature maintained at 37°C via a rectal feedback‐controlled heating pad (Harvard Apparatus).

### 2.3 Electrophysiological recordings in LC

Extracellular recordings were conducted using tungsten microelectrodes (3–4 MΩ impedance, FHC) that were slowly lowered into the burr hole 5.5–6.5 mm ventral from the top of the brain to reach LC (Paxinos & Watson, 2005). Ground and reference electrodes were inserted into the posterior neck muscles. Signals were amplified and filtered as previously described (Martlé et al., 2014). These filtered signals were digitized and sampled at 20 kHz using Spike2 9.09a (CED). LC units were identified during the experiment by characteristic firing rates (Hulsey et al., 2017; Martins & Froemke, 2015).

### 2.4 Experimental protocols (current determination, VNS paradigms)

**FIGURE 1:**
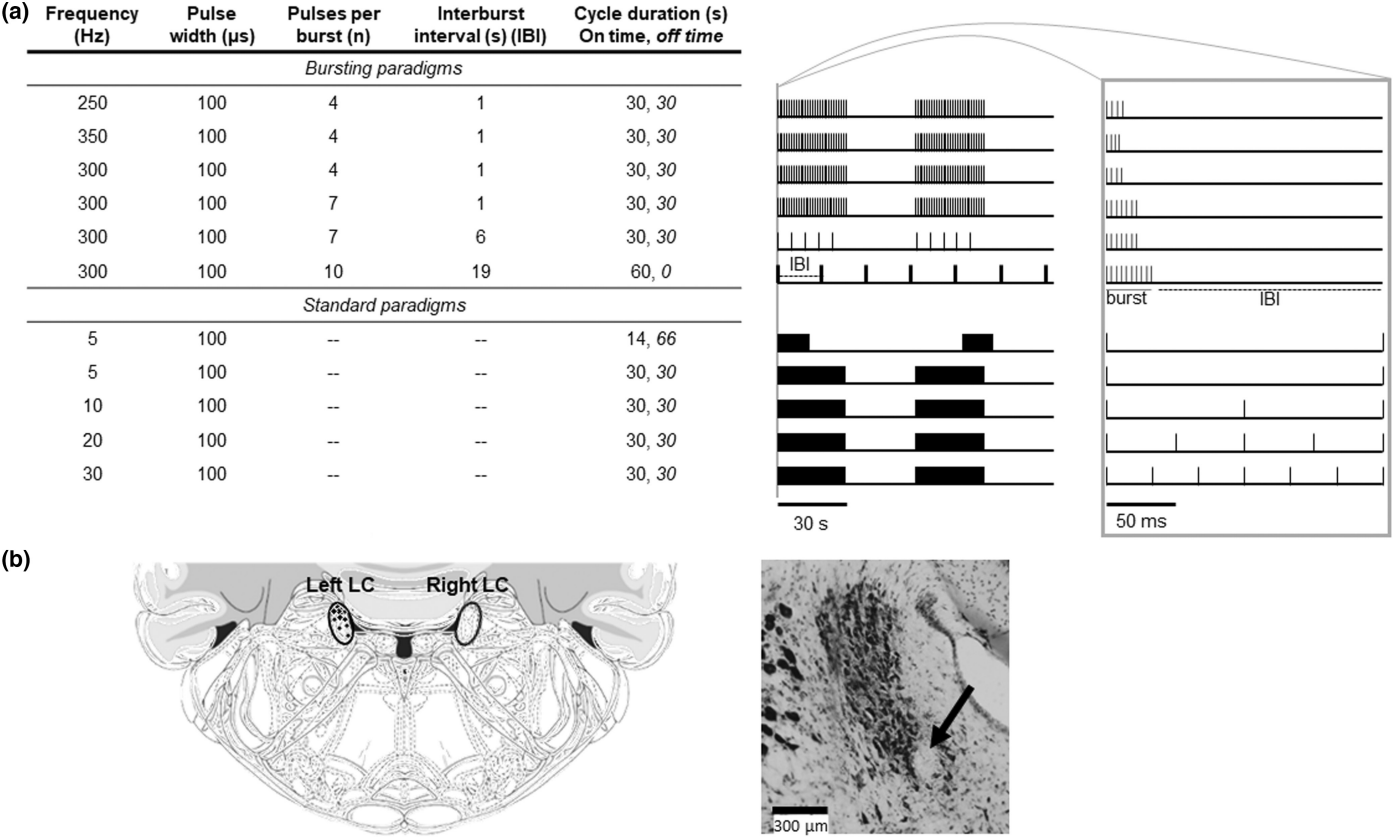
Experimental design. Table detailing all VNS paradigms tested (a: left), with bursting paradigms listed first followed by standard paradigms. Snapshots of 2 min window for each stimulation paradigm with tic marks denoting stimulation bursts (bursting) and blocks denoting stimulation “on time” (standard) showing two VNS cycles out of five total cycles delivered for each paradigm (a: middle). Gray bar shows magnification of 200 ms for each paradigm, with sample burst and interburst interval (IBI) delineated for the last bursting paradigm (a: right). Cresyl violet staining after electrolytic lesion confirmed electrode placement in left LC for each rat included in the study marked with separate black diamond on overlaid atlas images extending through the LC from Bregma (−9.4 to −10.1 mm) (b: right). A representative coronal section of LC is shown with a black arrow pointing at the electrolytic lesion (b: left).

Externalized lead wires from the cervical vagus nerve cuff were connected to a Master‐9 Programmable Pulse Stimulator and ISO‐Flex setup (A.M.P.I.) to test LC responsiveness to VNS paradigms. Intensity of VNS was determined for each rat based on the minimum current required to induce 5% physiological bradycardia during 20 Hz VNS as previously described (Beaumont et al., 2017) (termed bradycardic intensity, BI, 0.26 ± 0.06 mA). The recording electrode was slowly lowered toward LC using a micro manipulator (Scientifica). When the dorsal part of LC was reached, the electrode position remained fixed for approximately 5 min and then small dorsal‐ventral positioning adjustments were made to optimize the signal of external spontaneous action potentials to noise ratio (>3/1 ratio). Responsiveness of LC neurons to VNS was tested using a pre‐determined set of 11 VNS paradigms of varying frequency (5–350 Hz), bursting characteristics (4–10 pulses per burst, 1–19 s between bursts), and cycle duration (14–60 s) (Figure 1a). Each paradigm was delivered for approximately 5 min (5 VNS cycles) in a pseudorandom fashion, with at least a 2 min waiting period between paradigms. Once the VNS testing protocol was complete, the electrode was advanced by at least 100 μm to identify a new recording site. After the completion of 4 recording sites, an electrolytic lesion was created using 25 μA anodal current for 2 min to confirm the electrode position in LC (Figure 1b). Rats were then euthanized with 5% isoflurane and decapitated.

### 2.5 Histology

Brains were removed and post‐fixed in 10% formalin for 48 h, then transferred to 30% sucrose for >48 h for cryoprotection. Brains were frozen in OCT (Tissue‐Tek) and sectioned on a cryostat (Leica CM1850) at 40 μm. Tissue sections were stored at 4°C in PB with sodium azide. Sections were mounted on microscope slides and allowed to dry overnight. Tissue was stained with cresyl violet and cover slipped using Fisher Paramount Mounting Medium. An Olympus BX51 microscope was used to visualize the electrolytic lesion and confirm electrode placement at 10× magnification (Figure 1b) (Farrand et al., 2020).

### 2.6 Data analysis and statistics

#### 2.6.1 Spike sorting

The raw LC trace was first cleared of artifacts, mainly electrocardiogram contamination and stimulation artifacts during VNS. After artifact removal, principal component analysis (PCA) was performed in Spike2 to identify unique, single neuron signals based on shape, amplitude, and duration. PCA‐based template matching reliably identified consistent waveforms that were attributed to 1–4 individual neurons per recording site for these experiments (Beaumont et al., 2013, 2016). Using PCA‐identified neurons allowed determination of neuronal firing rates and changes in firing rate for single units relative to VNS paradigms.

#### 2.6.2 Strength and consistency of neuronal responses

**FIGURE 2:**
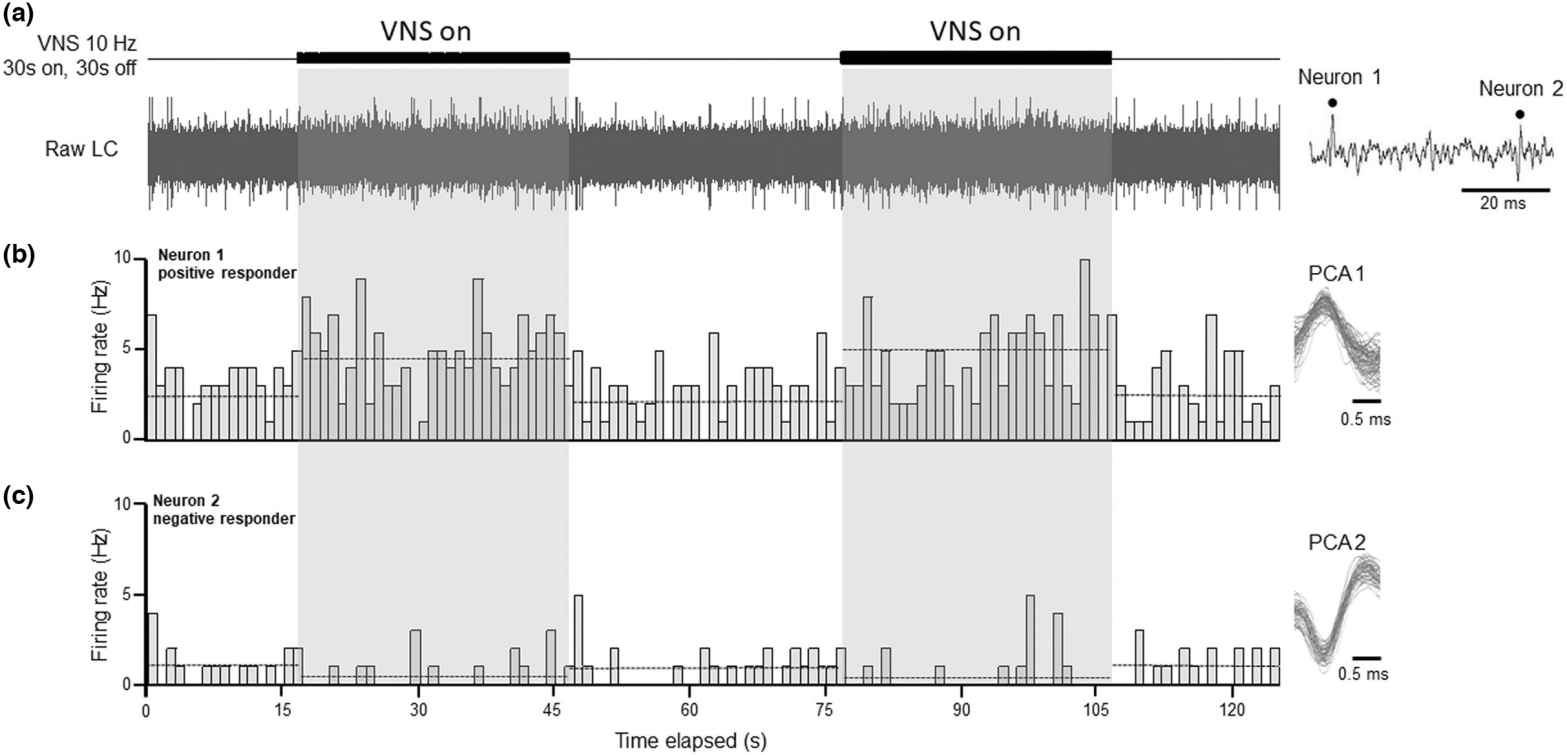
Exemplar neurons from each responder group. (a) The raw LC trace was subjected to principal component analysis (PCA) to identify individual neurons and an extended time scale inset on the right showed both recorded neurons. Neurons were isolated and classified based on changes in firing rate during VNS cycles, shown here to be 10 Hz, 30 s on, 30 s off (two of five VNS cycles shown here for clarity). Two neurons were identified from this example: a positive responder (b) neuron 1, 88% increase in firing rate during VNS 1 (4.7 Hz) compared to its baseline (2.5 Hz) and a negative responder (c) neuron 2, 38% decrease in firing rate during VNS 1 (0.34 Hz) compared to its baseline (0.7 Hz). Firing rates were determined and plotted in Hz based on the number of spikes observed per second for identified neurons. The PCA template for each neuron is shown on the right‐hand side with at least 50 matched spikes overlaid on each template.

Neurons were classified for strength analysis based on changes in firing rate during each VNS paradigm: negative responders (>15% decrease), non‐responders (−15 to +15% change) and positive responders (>15% increase) (Figure 2). Note that the baseline neuronal activity level had a stochastic profile with a constant value (<5% variation). The proportion of negative and positive responder neurons as well as the relative change in firing rate were assessed across 5 consecutive VNS cycles (mean + SEM) for 11 VNS paradigms using Prism9 (GraphPad). For overall analyses, 2×2 contingency tables were constructed using the number of positive or negative responder neurons versus VNS paradigms tested. Results were considered significant when *p* < 0.05, but statistical tests resulting in *p* < 0.1 were also reported when the number of degrees of freedom was limited (Mpofu et al., 2004).

For all paradigms and all neurons (922 combinations in total), the firing rates in the 11 chronological intervals (pre‐VNS, VNS 1 on, VNS 1 off, VNS 2 on, …, post‐VNS) formed an 11‐dimensional feature vector. The most prominent principal component of the resulting 922‐by‐11 matrix had the shape of a sawtooth, which motivated the use of cosine similarity to identify positive and negative responder neurons during VNS (Tan et al., 2005). Cosine similarity was defined as the angle between the feature vector and the sawtooth vector [−1, 1, −1, …, 1, −1]. Its theoretical value ranges from −1 (negatively consistent response) to +1 (positively consistent response). Cosine similarities were computed for each neuron and each paradigm, and were classified as negatively consistent (<−0.25), inconsistent (−0.25 to +0.25), or positively consistent (> + 0.25). The chosen threshold value corresponded to a qualitative change in behavior since very few of the neurons with subthreshold cosine similarity had a strong response to VNS.

#### 2.6.3 Synchrony between neurons

The interplay between neurons was quantified by Agmon's jitter‐based synchrony index (JBSI) (Agmon, 2012) with a coincidence window of 30 ms, as used in our previous VNS studies (Longpre et al., 2014; Salavatian et al., 2016). This index is independent of variations in firing rate and corrected for random coincidences, ranging between −1 (anti‐synchrony) and + 1 (perfect synchrony), a value of 0 meaning no synchrony. It enables calculation of a *p*‐value to establish that the null hypothesis of no synchrony can be rejected (Agmon, 2012). Since JBSI is asymmetric, ordered pairs of simultaneously active neurons (214 over all animals) were considered. JBSI during VNS was computed over the full VNS period (~5 min). Pre‐VNS intervals from all paradigms were pooled to calculate JBSI at baseline (~5 min). Synchrony was considered statistically significant when *p* < 0.001 (Longpre et al., 2014; Salavatian et al., 2016). When *p* ≥ 0.001, JBSI was set to zero. The changes in JBSI during each VNS paradigm compared to the pre‐VNS intervals were plotted, showing graded JBSI changes in the observed number of neuron pairs compared to pre‐VNS. JBSI were compared between VNS paradigms using pairwise *t*‐tests.

#### 2.6.4 Identification of a triggered response

The triggered response was identified for each vagal stimulation burst and the evoked action potential. The delay between the cervical vagus stimulation and the responses were approximately 50 ms. Smoothed histograms in the time window ±300 ms were created using Gaussian‐kernel density estimate and rescaled such that baseline density was 1.0. Delays from all neurons from all rats were combined in the histogram for each VNS paradigm. The resulting distribution of delays was modeled by a mixture of uniform (representing the background activity) and Gaussian distributions (triggered response) (Angelis et al., 2015). This Gaussian distribution was described by three parameters: the average delay (mean in ms), the jitter (standard deviation in ms) and the weight (percentage of triggered spikes within the time window shown in %). Hierarchical model Bayesian parameter estimation was performed using PyMC3 (Salvatier et al., 2016). Uncertainty in parameter estimates (credible interval) was assessed by the 95% highest posterior density interval (Gelman et al., 2013).

## 3 RESULTS

### 3.1 Initial responder neurons to VNS paradigms

The recording electrode was slowly lowered into the LC nucleus. When the stereotaxic coordinates were reached, the depth of the electrode was finely adjusted to maximize the neuronal signal to noise ratio (>3/1). When the positioning was done, the electrode was kept in place for at least 5 min, allowing the nervous tissue to stabilize around the tip of the electrode, which further improves the signal to noise ratio. A total of 85 neurons were recorded from 10 rats with a range of 7–10 neurons per rat. Two to three neurons were recorded per site and we allow a minimum of 100 μm between sites to exclude the possibility of picking up the same neuron twice. The spontaneous activity of LC neurons at baseline under α‐chloralose anesthesia ranged from 0.5 Hz to 12 Hz.

**FIGURE 3:**
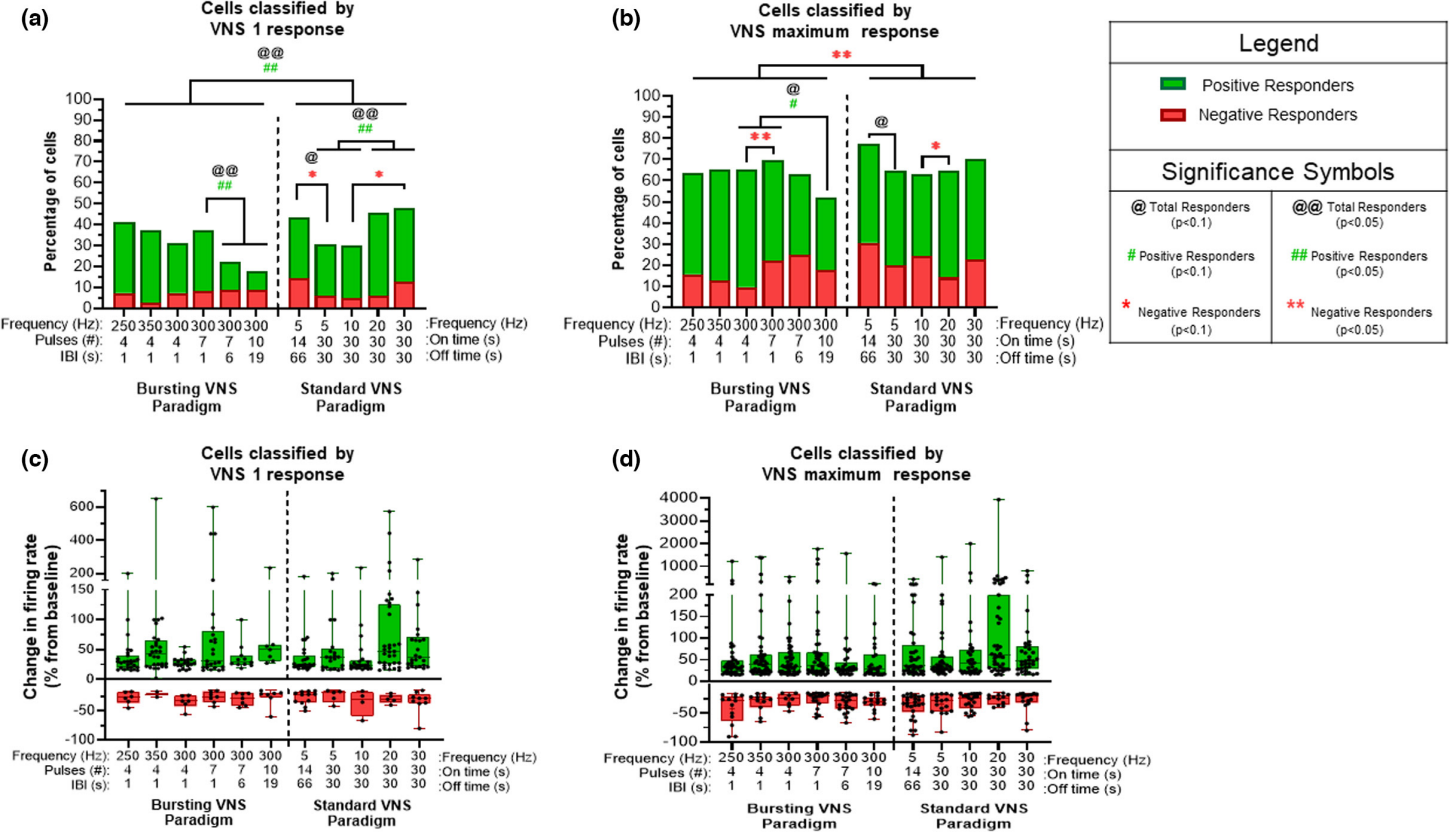
Magnitude firing rate changes across VNS paradigms in the LC. (a) The percentage of responder cells for each stimulation paradigm compared to their baseline level for VNS 1 is shown, with negative responders (>15% decrease), non‐responders (−15— + 15% change) and positive responders (>15% increase). For VNS 1, standard paradigms yield more positive and total responders than bursting paradigms (*p* < 0.05). For bursting paradigms at 300 Hz, increasing IBI to 6 s or 19 s or the pulse number from 7 to 10 decreased the number of total and positive responders (*p* < 0.05). While shorter on times during standard VNS drive more negative and total responders at 5 Hz (14 s vs. 30 s) (*p* < 0.1), higher standard frequencies (20/30 Hz) yield more positive and total responders than lower standard frequencies (5/10 Hz, *p* < 0.05). Additionally, negative responders are increased at 30 Hz compared to 10 Hz (*p* < 0.1). (b) The percentage of responder cells in each VNS paradigm were also classified according to their maximum response over the 5 VNS cycles. Note that no correlation was found of VNS response to any particular stimulation cycle. Overall, the standard paradigms have greater negative responders compared to bursting paradigms (*p* < 0.05). For standard paradigms, an “on time” of 30 s versus 14 s (5 Hz) showed fewer total responders (*p* < 0.1). Standard 20 Hz VNS yields decreased negative responders compared to 10 Hz (*p* < 0.1). For bursting paradigms at 300 Hz, increasing IBI to 19 s along with the pulse number from 4–7 to 10 decreased the number of total and positive responders (*p* < 0.1) and negative responders are increased with seven pulses compared to four pulses (*p* < 0.05). The boxplot graphs on the bottom row display 2.5, 25, 50, 75, and 97.5 percentiles of the relative strength for neuronal responses from their baseline activity during VNS 1 (c) and VNS Max (d). Although positive responders displayed a significant difference across all 11 paradigms, there were no significant post hoc results. No significant differences existed between paradigms for negative responders. ^@^ *p* < 0.1, ^#^ *p* < 0.1, *p < 0.1, ^@@^ *p* < 0.05, ^##^ *p* < 0.05, **p < 0.05.

To assess LC neurons' initial strength response to VNS, each recorded neuron was categorized into positive (Figure 2b), negative (Figure 2c) or non‐responder based on the percentage change in firing rate from baseline. A contingency analysis table was constructed based on the number of responders (positive or negative) for each VNS paradigm (11 total) and yielded overall significant differences during the first cycle (VNS 1) (*Χ* ^2^(30,*N* = 86 neurons) = 78.98, *p* < 0.0001). To determine where specific changes occurred, responders were grouped based on stimulation variables (standard vs. bursting, frequency, stimulation on time, number of pulses per burst, interburst interval (IBI)), followed by additional contingency analyses. During VNS 1, the total responders and positive responders were higher for standard paradigms compared to bursting paradigms (*p* < 0.05). Specifically for bursting paradigms at 300 Hz, increasing the IBI from 1 to 6 s or 19 s, or increasing the pulse number from 7 to 10 decreased the number of total and positive responders (*p* < 0.05). In regards to standard paradigms, shorter VNS on time (14 vs. 30 s) at 5 Hz had more negative and total responders (*p* < 0.1). For standard paradigms, higher frequencies (20/30 Hz) yield more positive and total responders than lower standard frequencies (5/10 Hz, *p* < 0.05). Additionally, negative responders are increased at 30 Hz compared to 10 Hz (*p* < 0.1) (Figure 3a). There were no significant changes in the magnitude of initial response across paradigms for any responder category (Figure 3c).

### 3.2 Responder neurons across five cycles of VNS

This analysis was used to determine the maximum response, either negative or positive, for each neuron over five cycles of VNS. Contingency analysis on the categorized responders (positive or negative) for each VNS paradigm (11 total) showed that significant changes existed (*Χ* ^2^(30,*N* = 86) = 56.73, *p* = 0.0023) (Figure 3b). Responders were grouped as described above to identify specific proportion differences between VNS paradigms. Note that no correlation was found of VNS response to any particular of the five stimulation cycles. It is important to note that the percent of neurons that showed a response over the five VNS cycles was significantly higher by about 2‐fold for the total, the positive and the negative responders, compared to their initial response at VNS 1 (*p* < 0.001). Indeed, repeated cycles of VNS showed a buildup effect over time. Overall, the standard paradigms had greater negative responders compared to bursting paradigms (*p* < 0.05). For standard paradigms, an on‐time of 30 s versus 14 s (5 Hz) showed fewer total responders (*p* < 0.1). Standard 20 Hz VNS yields decreased negative responders compared to 10 Hz (*p* < 0.1). For bursting paradigms at 300 Hz, increasing IBI at 19 s along with the pulse number from 4–7 to 10 decreased the number of total and positive responders (*p* < 0.1) and negative responders were increased with seven pulses compared to four pulses (*p* < 0.05) (Figure 3b). Due to the variation in each neuronal response, no significant change was detected in the magnitude of maximum response across paradigms for any responder category (Figure 3d).

### 3.3 Consistency between LC neuronal activity and cycles of VNS

**FIGURE 4:**
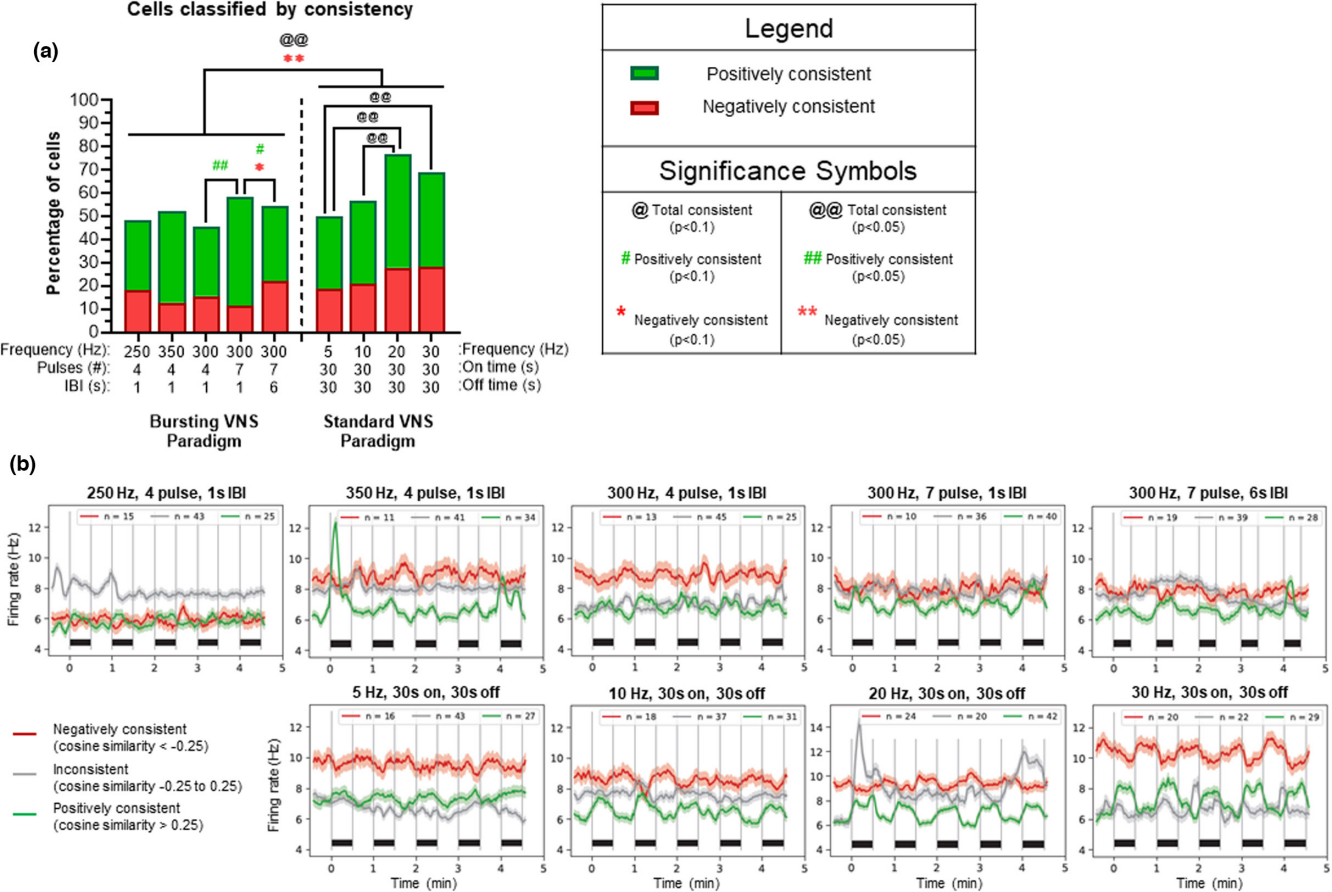
Consistency between LC neuronal activity and cycles of VNS. (a) Neurons were classified according to consistency (i.e., correlation of LC activity to a cosine mathematical curve corresponding to cycles of VNS). The percentage of neurons that increased or decreased their activity during VNS “on time” were categorized as positively (green) and negatively (red) consistent for each paradigm. Overall, standard paradigms have more total and negatively consistent neurons than bursting paradigms (*p* < 0.01). Standard VNS at 20 Hz yields more total consistent neurons than 5 Hz or 10 Hz (*p* < 0.05) and standard VNS at 30 Hz yields more total consistent neurons than 5 Hz (*p* < 0.05). For bursting frequencies, the number of positively consistent neurons is higher at seven pulses compared to four pulses (*p* < 0.05). Longer interburst intervals (IBIs) yield fewer positively consistent and more negatively consistent neurons (1 s vs. 6 s, *p* < 0.1). (b) Remaining graphs show continuous evolutions of firing rates that were computed and plotted using a 5 s sliding window for negatively consistent (red), inconsistent (gray), and positively consistent (green) neurons across the five VNS cycles delivered for each bursting (b: top) and standard paradigm (b: bottom) with the number of cells for each category shown at the top of each graph. Note that each black bar above the *x*‐axis represents the VNS “on time” of 30 s. ^@^ *p* < 0.1, ^#^ *p* < 0.1, *p < 0.1, ^@@^ *p* < 0.05, ^##^ *p* < 0.05, **p < 0.05.

Evaluation of changes in spontaneous firing rate, while important, is only one component of how VNS can alter the neuronal activity in LC. Therefore, neurons were classified not only according to strength of firing rate changes, but also according to consistency of changes in regards to VNS cycles. Principal component analysis of firing rates revealed a sawtooth profile with peaks during VNS “on time” and troughs during “off time”. Significant changes in overall contingency analysis were observed using this cosine‐similarity‐based classification (*Χ* ^2^(16, *N* = 86) = 36.34, *p* = 0.0026). (Figure 4a). The changes for each neuronal category (positively consistent, negatively consistent or inconsistent) for each VNS paradigm are shown over five cycles of VNS (Figure 4b). Standard paradigms had more total and negatively consistent neurons than bursting paradigms (*p* < 0.05). Standard 20 Hz yielded more total neurons synchronized to VNS than lower frequencies (5/10 Hz, *p* < 0.05) and 30 Hz yielded more total neurons synchronized than 5 Hz (*p* < 0.05). For bursting paradigms at 300 Hz, the number of positively consistent neurons increased with the number of pulses delivered (4 vs. 7 pulses, *p* < 0.05). At 300 Hz, the longer IBI (from 1 s to 6 s) yielded fewer positively consistent and more negatively consistent neurons (*p* < 0.1). Note that the VNS parameters at (1) 300 Hz, 10 pulse, 19 s IBI and (2) 5 Hz,14 s on, 66 s off were excluded from this analysis because the stimulation on/off periods did not match.

### 3.4 Integration of strength and consistency of the neuronal responses

**FIGURE 5:**
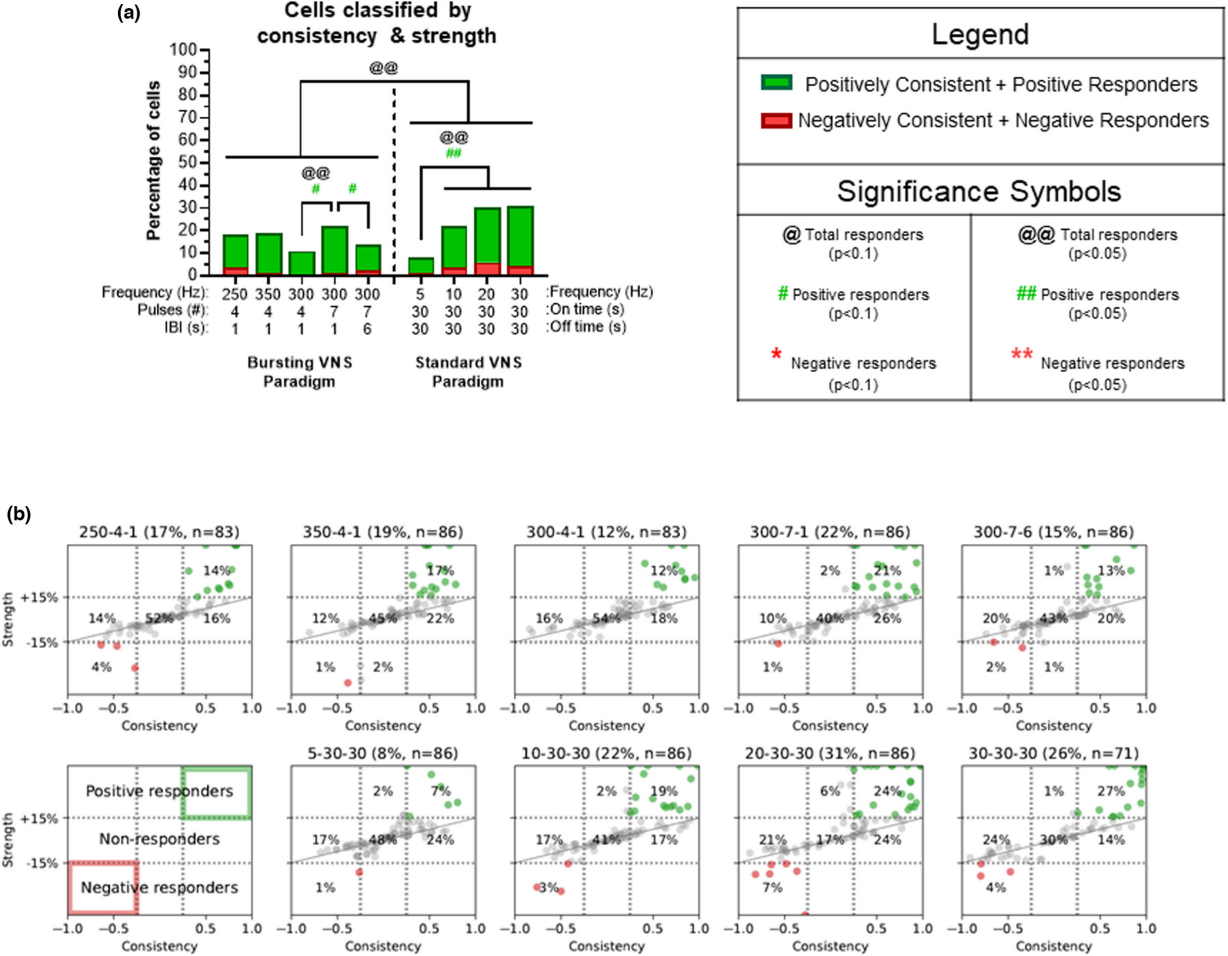
Integration of consistency and magnitude of the neuronal responses. (a) Neurons that are both positively consistent and positive responders are shaded in green, and neurons that are both negatively consistent and negative responders are shaded in red. Percentages of neurons shown in the red and green are plotted for each paradigm. Overall, standard paradigms have more total consistent/strong neurons than bursting paradigms (*p* < 0.05). Analysis of standard paradigms indicated that 5 Hz has fewer total consistent/strong neurons and fewer positively consistent/strong neurons than higher frequencies (10/20/30 Hz, *p* < 0.05). In regards to bursting VNS at (300 Hz, seven pulses, 1 s IBI) the number of total (*p* < 0.05) and positively consistent/strong (*p* < 0.1) neurons was higher compared to four pulses. Increasing the interburst interval (IBI) from 1 to 6 s decreased the proportion of positively consistent/strong neurons. Negatively consistent/strong neurons could not be evaluated alone due to small numbers of neurons displaying these characteristics. (b) These graphs show the strength versus consistency plots for each bursting (b: top) and standard (b: bottom) paradigm analyzed over five cycles of VNS and these graphs were used to generate the graph bars (a). ^@^ *p* < 0.1, ^#^ *p* < 0.1, *p < 0.1, ^@@^ *p* < 0.05, ^##^ *p* < 0.05, **p < 0.05.

The relationship between consistency and strength of the neuronal responses were linked together as positively consistent + positive responders or negatively consistent + negative responders for all paradigms. The percentage of cells in each category are plotted for each paradigm (Figure 5a). Negatively consistent/strong neurons could not be evaluated alone without violating sufficient sample size rules for contingency analysis; therefore, only total consistent/strong neurons and positively consistent/strong neurons were compared across VNS paradigms. Significant overall changes existed using these classification parameters (*Χ* ^2^(8,*N* = 86) = 26.10, *p* = 0.0010). Overall, standard paradigms had more consistent/strong neurons than bursting paradigms (*p* < 0.05). Analysis of standard paradigms indicated that 5 Hz has fewer total and positively consistent/strong neurons than higher frequencies (10/20/30 Hz, *p* < 0.05). For bursting frequencies at 300 Hz, the number of pulses per burst (4 to 7) increased the number of total (*p* < 0.05) and positively (*p* < 0.1) consistent/strong neurons, while a longer IBI (1 s vs. 6 s) decreases the number of positively consistent/strong neurons (*p* < 0.1). As with the previous figure, 300 Hz, 10 pulse, 19 s IBI and 5 Hz, 14 s on, 66 s off were excluded from this analysis.

### 3.5 Interneuronal synchrony across paradigms

**FIGURE 6:**
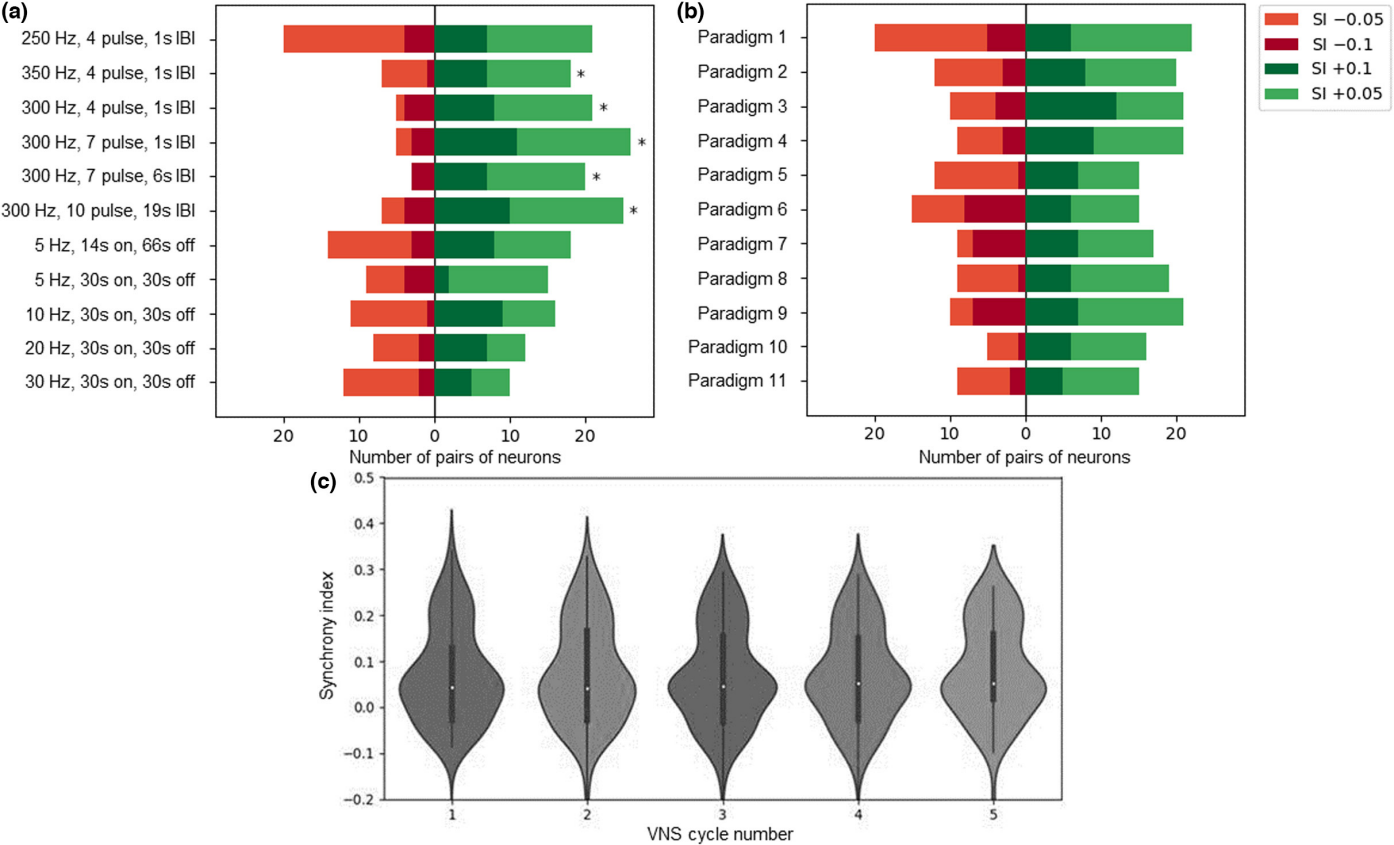
Interneuronal synchrony across paradigms. Synchrony indices (SI) independent of firing rate changes were computed using ordered pairs of simultaneously recorded neurons. These have been corrected for possible random coincidences within a 30 ms window and normalized between −1 (anti‐synchrony) and + 1 (perfect synchrony). SI was determined for all pairs of neurons during the pre‐VNS baseline period and during each VNS paradigm. Only SI with *p* < 0.001 were considered due to the large sample size (214 pairs). (a) The changes in SI during each VNS paradigm compared to the pre‐VNS intervals are plotted, showing graded SI changes in the observed number of neuron pairs. SI were increased during VNS for all 300 and 350 Hz bursting paradigms (*p < 0.05). (b) SI changes during VNS are plotted for each paradigm according to the order in which they were applied. No significant cumulative effects were observed across time. (c) SI changes during VNS are shown for each VNS 1–5 separately. The median value is shown as a white dot with black boxes denoting 25%–75% quartile ranges and the whisker showing the total range of values. Paradigms were pooled for this analysis to ensure appropriate sample size. No significant changes were observed between VNS 1–5.

Synchrony indices (JBSI) were determined based on the timing of action potentials between pairs of neurons that were recorded simultaneously. Pairwise *t*‐tests indicated that synchrony was increased only for all 300 and 350 Hz bursting VNS paradigms (*p* < 0.05), in comparison to their synchrony status during baseline (Figure 6a). Next, synchrony changes were in independent cumulative order in which VNS paradigms were delivered (Figure 6b). Also, no overall cumulative effects on synchrony over VNS1‐5 were found (Figure 6c). These results suggest that observed changes in synchrony are an effect of the paradigm itself rather than the order in which paradigms were delivered.

### 3.6 Evaluation of triggered responses in LC using bursting VNS

**FIGURE 7:**
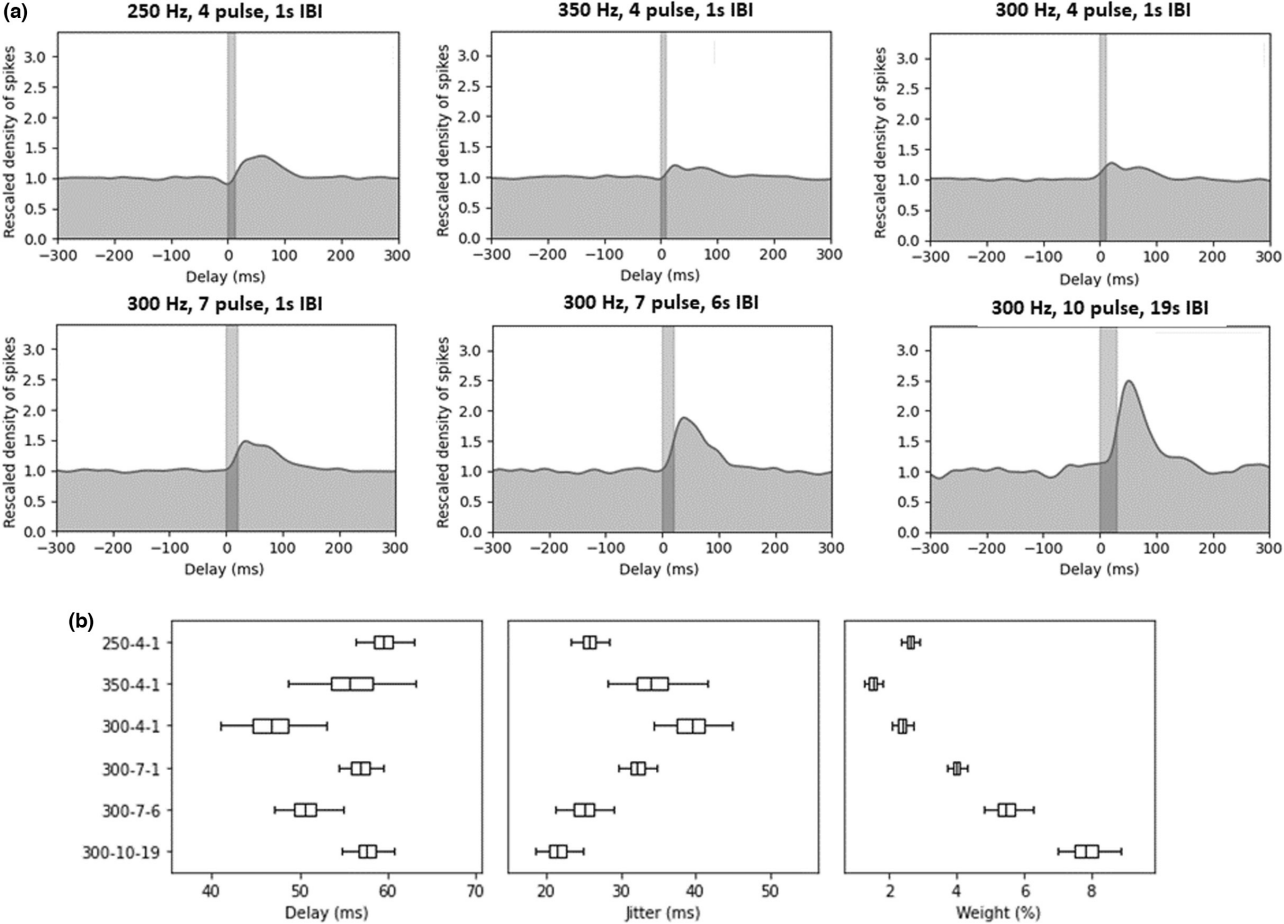
Evaluation of triggered responses in LC using bursting VNS. A spike density analysis was conducted to assess direct responses for each bursting paradigm. Baseline firing was rescaled to 1.0 across the 300 ms preceding pulse bursts. Bayesian parameter estimation was used to plot the direct response for each paradigm as a Gaussian distribution. While frequency does not have a large effect on the triggered response (a: top), higher numbers of pulses per burst and longer interburst intervals (IBIs) drive an increased triggered responses in the LC (a: bottom). (b) All paradigms show a similar delay (45–60 ms), with higher number of pulses and longer IBIs showing reduced jitter and higher weight. Further breakdown of weight assessment shows spikes per burst and spikes per stimulus. All boxplot graphs on the bottom row display 2.5, 25, 50, 75, and 97.5 percentiles for each paradigm. This direct response analysis was only conducted for bursting paradigms since standard paradigms at higher frequencies (20/30 Hz) have consecutive pulses overlapping the expected 45–60 ms delay window.

Triggered response analyses were conducted on bursting paradigms only since the expected delay for evoked responses in LC (45–60 ms) overlaps with continuous standard VNS pulses delivered at 5–30 Hz. While frequency did not seem to have a large effect on the direct responses, higher numbers of pulses per burst and longer IBIs drove an increased probability of a triggered response in the LC (Figure 7a). All paradigms showed a similar delay (45–60 ms), with higher number of pulses and longer IBIs showing reduced jitter and higher weight (Figure 7b). In this analysis, the weight is interpreted as the percentage of spikes identified as direct responses. Error bars correspond to Bayesian credible intervals. Non‐overlapping credible intervals indicate that there is a 95% chance that the parameter is different from the other paradigms. We can then assume that 300 Hz/10 pulse/19 s IBI > 300 Hz/7 pulse/6 s IBI > all the other bursting VNS paradigms.

## 4 DISCUSSION

The LC is considered to be a key mediator of VNS effects in the brain (Dorr & Debonnel, 2006; Furmaga et al., 2011; Grimonprez et al., 2015; Groves et al., 2005). Responses to acute VNS in the current study induced reliable changes in LC single unit activity. The order in which the 11 VNS paradigms were delivered did not affect the results, which implies causation of the VNS paradigm itself rather than a build‐up of overall stimulation effects. The current study was designed to mimic potential clinical parameters. Indeed, all VNS paradigms were done at the same intensity, which was defined previously by our group as the bradycardic threshold (BI) (Beaumont et al., 2017; Cooper et al., 2021). The use of BI provided a physiological benchmark that allowed for standardization of current delivery across animals by adjusting for the contact between the vagus nerve and the bipolar cuff electrodes. Moreover, we have previously shown that bradycardic intensity only activated vagal myelinated A‐fiber afferents, since evoked monosynaptic inputs were only detected in NTS neurons receiving these myelinated vagal afferents (Beaumont et al., 2017). Previous studies have examined the effects of VNS on LC firing and showed that using a larger pulse width and higher current intensities elicited higher LC neuronal activity (Hulsey et al., 2017). Higher intensity/larger pulse width would recruit additional vagal afferents and potentially a proportion of C‐fiber afferents, leading to greater activation of NTS neurons and upstream nuclei including the LC. Therefore, this study examined the impact of VNS parameters (namely frequency and bursting characteristics including number of pulses per burst and interburst intervals) using a constant intensity on the LC activation properties. It cannot be excluded that the activation of vagal efferent fibers during VNS could also induce reflexive changes in afferent activity.

The LC neurons were categorized as responders when VNS modified their activity level by 15% compared to baseline. Responder neurons for the first cycle (VNS 1) were present in ~35% of neurons, but then increased throughout the following four VNS cycles to ~65%, but it was not possible to associate that increase in activity to any particular cycles of VNS 2–5. That signal amplification was present for all VNS parameters studied. We have previously shown that VNS at 20 Hz produced an amplification effect over five VNS cycles in ~20% of NTS neurons. Interestingly, all of these NTS neurons were polysynaptically innervated from the vagus and none of them were monosynaptically innervated (Cooper et al., 2021). From these evidences, it is likely that this amplification effect is generated throughout the CNS network and some particular VNS settings are most likely beneficial for promoting this neuronal amplification over multiple VNS cycles. Moreover, future studies exploring longer VNS periods (hours to weeks) are needed to better understand their effect on LC neurons.

### 4.1 Standard VNS paradigms

For standard paradigms, the increased activity in combination with the consistency of the response for each cycle of VNS was higher for 20–30 Hz compared to 5 Hz. These findings align with clinical data suggesting that 20–30 Hz works best for activating nuclei in the brain and better reduce clinical symptoms for both epilepsy and depression (Rush et al., 2005; Sackeim et al., 2007; Yamamoto, 2015). Alternatively, low VNS frequencies (1–10 Hz) have demonstrated greater efficacy in the clinic for peripheral conditions including rheumatoid arthritis and gut motility (Koopman et al., 2016; Lu et al., 2018). Currently, it is thought that low frequency VNS works in these disorders by increasing the parasympathetic drive to visceral organs directly (Bonaz et al., 2017).

### 4.2 Bursting VNS paradigms

While very little research exists examining LC activation with such high‐frequency VNS parameters, a previous study suggested that increasing the stimulation frequency affected the timing of LC activation, but not the magnitude of the response (Hulsey et al., 2017), though this group did not account for the vast differences in responder classes (positive vs. negative responders) observed in the current study. Looking at alternative methods of brain stimulation such as transcranial magnetic stimulation, bursting paradigms have shown to be more beneficial than traditional repetitive paradigms both in tolerability as well as increased cortical excitability (Han et al., 2018; Ni et al., 2014). In the current study, the bursting parading 300 Hz, seven pulses and 1 s IBI yield more positively consistent + positive responders compared to a smaller number of pulses (Yuan & Silberstein, 2015a) or longer IBI (6 s). Interestingly, when looking at the triggered responses, which was the number of spikes/burst (weight) recorded at a latency of 50 ms, the highest spike densities were reported with more pulses per burst (10 > 7 > 4) and longer IBI (19 > 6 > 1 s). This is counterintuitive, because in a simple neuronal system, the positive activation of LC neurons with VNS should normally be greater with a larger triggered response (weight) provided from vagal afferents, but the opposite phenomenon is observed. Since synchronized inputs are sent to LC neurons during VNS, instead of a normal stochastic input from vagal afferents, the data suggests that shorter IBIs and lower number of pulses better positively activate LC neurons. We can speculate that these VNS parameters provide a greater amplification of the signal through the complex mammalian neuronal network (Beaumont et al., 2017).

### 4.3 Standard versus bursting VNS frequencies

Previous studies in preclinical epilepsy and Parkinson's disease models have suggested that bursting VNS may be more tolerable and, in some cases, provide greater benefits over standard paradigms (Alexander & McNamara, 2012; Farrand et al., 2020; Martlé et al., 2014; Szabó et al., 2017). This could indicate a change in the basal firing rate of single cells driven by either increased inhibitory input or reduced excitatory input from the solitary nucleus or paragigantocellular nucleus (Lopes et al., 2016). However, because input from both of these regions to LC is thought to be largely excitatory, and VNS is known to increase firing in the solitary nucleus (Beaumont et al., 2017), it seems more likely that activation of either microcircuits of LC neurons or activation of higher regions such as the paraventricular hypothalamic nucleus (PVN) creates a negative feedback loop to slow firing of these LC neurons with VNS. Indeed, a small subset of LC neurons has been shown to co‐release GABA and provide inhibitory influence over local LC populations (Breton‐Provencher & Sur, 2019). Additionally, peptidergic projections from the PVN to the LC have been demonstrated (Reyes et al., 2005). It is possible that standard paradigms are able to more directly target these circuits while bursting paradigms differentially activate excitatory circuits, though future studies investigating the contribution of each of these pathways is warranted.

### 4.4 Synchrony in LC firing

Another important component of VNS effects in LC is the synchrony of recorded neurons during stimulation compared to their spontaneous activity at baseline. This study showed that the synchrony between pairs of neurons was not increased with all standard VNS frequencies (5–30 Hz). Alternatively, all of the bursting paradigms at 300 and 350 Hz showed increased synchrony. These observed data further support the idea that bursting frequencies above 300 Hz better activate linked nuclei in the central nervous system (Hassert et al., 2004; Roosevelt et al., 2006). Indeed, standard paradigms trend toward more dichotomous effects on synchrony. Increased synchrony during bursting VNS suggests common input/network recruitment that may be ideal for nuclei like LC with strong phasic response profiles (O'Donnell et al., 2012). Natural bursting LC responses to phasic input may help explain the increased synchrony in LC during bursting VNS.

## 5 CONCLUSIONS

Differential activation profiles are present in LC depending on the type of VNS delivered, though it is currently unclear if this is linked to endogenous neuronal attributes or to population/network level organization. The current data suggest that VNS settings can be optimized depending on the clinical goal. For example, if LC output needs to be increased as with Parkinson's disease, depression, or epilepsy, settings inducing positive responses may increase clinical benefits. Future studies looking at chronic VNS effect on LC activation are also necessary to draw definite conclusions on the clinical efficacy.

## AUTHOR CONTRIBUTIONS

Ryan Verner and Eric Beaumont conceptualized, helped develop methodology, provided resources, and acquired funding for this project. Ariana Farrand, Ryan Verner, and Eric Beaumont were responsible for project administration and investigation. Formal analysis, software, data curation, and data visualization were conducted by Ariana Farrand, Misty Owens, Vincent Jacquemet, and Eric Beaumont. The original draft was prepared by Ariana Farrand and Eric Beaumont. All authors participated in review and editing of the manuscript.
